# Failed septal extension graft in a patient with a history of radiotherapy

**DOI:** 10.1186/s40902-016-0086-9

**Published:** 2016-10-25

**Authors:** Il Gyu Kang, Seon Tae Kim, Seok Ho Lee, Min Kwan Baek

**Affiliations:** 1 Department of Otolaryngology-Head and Neck Surgery, Graduate School of Medicine, Gachon University of Medicine and Science, Gil Medical Center, Guwoldong 1198, NamdongGu, Incheon City, 405-760 Korea; 2 Department of Radiation Oncology, Graduate School of Medicine, Gachon University of Medicine and Science, Gil Medical Center, Incheon, Korea

**Keywords:** Nasal septum, Rhinoplasty, Radiotherapy, Nasal cartilages

## Abstract

**Background:**

This report describes the authors’ experience of “melting” septal cartilage after placement of a septal extension graft in a nasopharyngeal cancer patient that had been previously undergone radiation therapy, and provides a review of the literature.

**Methods:**

Electronic medical records were used to obtain details of the patient’s clinical history.

**Results:**

A 32-year-old woman, who had previously undergone radiotherapy for nasopharyngeal cancer, visited our department to for rhinoplasty. Rhinoplasty was performed using a septal extension graft to raise the nasal tip (first operation). Five days after surgery, it was found that the septal extension graft was melting without any signs of infection, that is, the graft had softened, lost elasticity, thinned, and partially disappeared without any sign of infection at 5 days, and thus, the nasal tip was reconstructed with conchal cartilage (second operation). Five months after surgery, it was found that almost all septal cartilage had disappeared without any sign of infection, and thus, the entire nasal septum was reconstructed using 2-mm costal cartilage and an onlay graft was used for tip augmentation (third operation).

**Conclusions:**

After cartilage has been exposed to radiotherapy, its patency should be viewed with suspicion. Further studies are needed for determine the mechanism responsible for cartilage damage after radiotherapy.

## Background

Radiotherapy has been shown to be effective for controlling carcinoma of the larynx and nasopharynx, and combined radiotherapy and an organ-sparing surgical protocol has been frequently used to treat patients with advanced-stage laryngeal or nasopharyngeal carcinoma. However, radiation therapy may cause hypoxic, hypovascular, and hypocellular changes that impair normal collagen synthesis and cell production and lead to tissue breakdown and the formation of chronic non-healing wounds [1]. Nevertheless, nasal cartilage necrosis is not common after radiotherapy in nasopharyngeal cancer patients.

We review a case of “melting” septal cartilage after septal extension graft placement in a nasopharyngeal cancer patient who had received radiotherapy prior to the graft placement.

## Case presentation

**Fig. 1:**
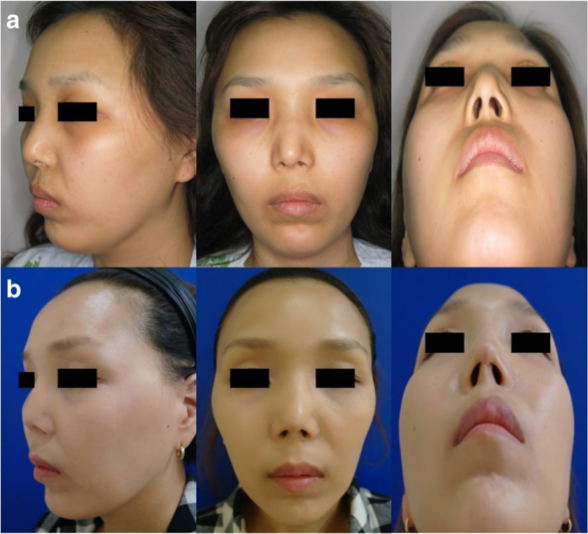
**a** Preoperative evaluation prior to initial rhinoplasty demonstrating drooping of the nasal tip, wide nasal width, and nasal septal deviation. **b** Postoperative photos taken after third operation demonstrating a corrected nasal tip projection and a narrower nasal width

**Fig. 2:**
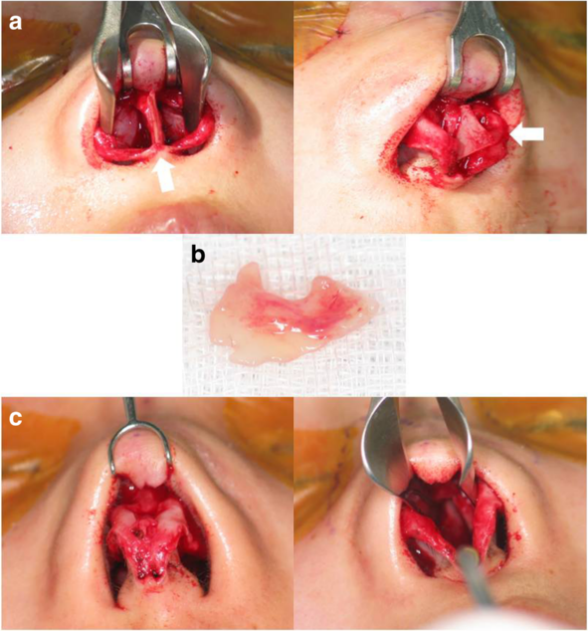
**a** This figure shows a nasal septal extension (*white arrow*) graft in the operation field (first operation). **b** At the second operation conducted 5 days after the first operation, the septal extension graft appeared to be “melting” without any sign of infection, that is, grafted cartilage had softened, lost elasticity, thinned, and partially disappeared in the absence of a foul odor, purulent discharge, or erythematous tissue swelling. **c** Five months after the second operation, septal cartilage had almost disappeared without any sign of infection (third operation)

A 32-year-old woman, who had undergone radiotherapy for nasopharyngeal cancer 4 years previously, visited our department to undergo rhinoplasty. Observation revealed slight dropping of the nasal tip, a wide nasal width, and the presence of nasal septal deviation (Fig. 1a). Under general anesthesia, the columella was incised using an inverted V incision. In addition, the incision was connected with a marginal incision and then dissection was performed over the perichondrial plane. A 1.5 × 1.5 cm-sized piece of septal cartilage was removed, while preserving the L-strut, which was ~1.5 × 1.5 cm sizes, and then medial and lateral osteotomy were performed bilaterally to reduce nasal width. In addition, septal extension grafting (type III) was performed using septal cartilage, which was fixed on the septal end using PDS 4-0. The lower lateral cartilage was placed in a high position and fixed it to the septal extension graft using PDS 4-0 (Fig. 2a). The nasal dorsum was also raised by ~4 mm using processed fascia lata. After the procedure, the patient was satisfied with the resulting heightened nasal tip.

However, from the third day after surgery, the nasal tip became progressively depressed, and thus, on the fifth day after surgery, the incision site was opened. It was found that the septal extension graft appeared to be “melting,” that is, the cartilage had softened, lost elasticity, thinned, and partially disappeared without any sign of infection, such as a foul odor, purulent discharge, or erythematous tissue swelling (Fig. 2b). Accordingly, the nasal tip was reconstructed with conchal cartilage. However, the nasal tip gradually and progressively became depressed and showed caudal rotation. Accordingly, a revision operation using costal cartilage was performed at 5 months after the second operation. Briefly, the incision site was reopened and the entire nasal septum was found to have almost disappeared without any sign of infection (Fig. 2c). The septum was reconstructed with 2-mm costal cartilage, and an onlay graft was used for tip augmentation. After this third operation, we investigated the cause of the septal cartilage “melting,” based on the presumption that it was associated with the previous radiation therapy. A review of radiotherapy details showed that the nasal septum had been included in the irradiated field and that a 6-megavoltage (MeV) machine had been used five times a week to administer a bilateral opposing RT (radiotherapy) field to a total of 68 gray (Gy) in 36 fractions from the nasopharynx to middle cervical lymph nodes. The patient was partially satisfied with the operative result at 1 year after the third operation (Fig. 1b).

### Discussion

The immediate and late complications of radiotherapy are soft-tissue necrosis, xerostomia, mucositis, osteoradionecrosis, and chondroradionecrosis [2]. In children, growing cartilage is particularly radiosensitive; 10 Gy (1000 rad) can slow growth and growth deficits are irreversible at ~20 Gy [3]. In a pathologic study by Keene et al. on human laryngeal specimens, the overall incidence of chondroradionecrosis was 26 %, although only 3 % were clinically meaningful [4]. Chondroradionecrosis has been showed to be related to total radiation dose, duration of therapy, number of fractions delivered, and field size [5]. Our patient did not have chondroradionecrosis and septal cartilage remained intact during surgery. We assume that the septal cartilage “melted” because radiotherapy and/or the dissection plane under perichondrium used to obtain septal cartilage compromised its blood supply. It has been shown that the obliteration of capillaries by exposure to ionizing radiation can reduce blood supply to laryngeal and tracheal cartilage and lead to cartilage malnutrition [6]. Furthermore, although we consider it less likely, it is also possible that nasal septal cartilage destruction occurred during tumor regression induced by concurrent chemoradiation therapy (CCRT).

Hugenberg et al. reported that articular cartilage from adult humans or large animal species appears to degrade after exposure to radiation [7]. This response is characterized by active degradation of cartilage matrix and reduced proteoglycan production in pigs, dogs, and man [7, 8]. Furthermore, collagen II synthesis has been shown to be reduced in articular chondrocytes harvested from a large animal species exposed to radiation [9], and if radiation alters cartilage matrix metabolism, including the active degradation of proteoglycans or lowered proteoglycan or collagen II synthesis, reductions in the compressive modulus of irradiated cartilage would be expected. Lindburg et al. studied the effects of low doses of X-ray radiation on porcine articular cartilage explants and found that irradiation affects the bulk mechanical properties of cartilage as well as superficial characteristics [10]. We presume that the septal cartilage melted due to these factors, namely, radiotherapy-induced insufficiency of blood supply, degradation of cartilage matrix, reduced proteoglycan production, and reduced collagen II synthesis. We regret that no histopathologic study was performed on the melting cartilage.

## Conclusions

After cartilage has been exposed to penetrating radiation, it should be approached cautiously if surgery is required. Further studies are needed to identify the mechanism responsible for cartilage damage after radiotherapy and to determine whether radiotherapy causes long-term cartilage damage.
